# The influence of gender on the primary care management of diabetes in Tunisia

**Published:** 2009-08-22

**Authors:** Hugh Alberti, Benjamin Alberti

**Affiliations:** 1 School of Medicine & Health, Durham University, Queens Campus, Stockton, United Kingdom,; 2 Department of Sociology, Framingham State College, 100 State Street, Framingham MA, USA

**Keywords:** Diabetes care, gender, quality of care, Tunisia

## Abstract

**Background:**

Gender differences in access to high quality care for chronic illnesses have been suggested yet little work in this potentially vital area of health care inequality has been undertaken in Africa. We explored the influence of patient gender on the care of people with diabetes within a multi-method, national study of diabetes management in primary care in Tunisia.

**Methods:**

Methodologies used were quantitative (nationwide randomized study of 2160 medical records) and qualitative (participant observation, focus groups and interviews of patients and health care professionals).

**Results:**

Differences in patient characteristics, treatments prescribed, process and outcome data and access to care variables were demonstrated. The most striking disparity found was the high female to male ratio of patients attending for diabetes care (61.1%). A number of possible explanations for this emerged: Men were thought to under-attend for practical, financial and behavioural reasons whereas women were thought to have increased morbidity and potentially over-attend for social and psychological reasons.

**Conclusion:**

We have demonstrated a number of disparities in the care of men and women with diabetes in Tunisian primary care. In particular, a dual but related problem emerges from the data: more women than men attend for diabetes care and yet women do not get the same level of risk factor control as men. A number of local explanations for these disparities have emerged, which inform our analysis of the impact of gendered beliefs on diabetes care. Strategies to address these disparities will require a careful consideration of local beliefs and practices.

## Background

Diabetes mellitus is a chronic disorder that is assuming epidemic proportions. There are nearly 200 million people with diabetes and this figure has been predicted to rise to 366 million by 2030 [1]. The developing world in particular is facing a major epidemic. The increase in prevalence is predicted to approach 200% in developing countries compared to 45% in developed countries, due to population ageing, urbanisation, unhealthy diets, obesity and a sedentary lifestyle. The highest increases in prevalence of diabetes are anticipated to be in the Middle East and North African regions [1]. Providing effective quality of care to patients with diabetes is essential as good control of blood pressure and glucose significantly reduces the risk of cardiovascular and microvascular complications [2, 3].

Gender differences in access to high quality care for chronic illnesses have been demonstrated [4]. The effect of gender on glycaemic control is less conclusive but there appears to be increasing evidence that women have poorer quality of care than men [5, 6]. Yet women appear to attend health care facilities more often than men worldwide [7–10]. Little work in this potentially vital area of health care inequality has been undertaken in the Middle East or North Africa.

In Tunisia, the prevalence of diabetes mellitus has significantly increased over recent decades to 10% of adults [11]. In response, the Tunisian Ministry of Health has initiated a national program to improve the management of hypertension and diabetes in primary care. The overall objective is to ensure quality, standardised and regular care in order to reduce complications [12]. The program includes training of primary health care doctors, provision of essential medicines and public health education. Primary care health centres have also been encouraged to introduce weekly, chronic disease clinics and to use disease-specific medical records for patients with diabetes and hypertension.

Although patients with diabetes in Tunisia can choose to attend either a primary or secondary care doctor in either the public or private sector, the majority of patients nationwide are managed in public sector; primary care health centres (PHCCs). The exact proportion of people who attend PHCCs in comparison to other facilities is difficult to ascertain due to the lack of statistical data from the private sector, but in general, the poorer sections of society attend public sector facilities. Most health care provision in Tunisia is heavily subsidized. Those in employment pay for health insurance, which covers most, but not all, of health expenses within the public sector and some aspects of the private sector. Families out of employment classify for either free care or subsidized care. As part of a wider, national, qualitative/quantitative study on diabetes care in PHCCs [13, 14], we explored the influence of patient gender on the care of people with diabetes in Tunisia and particularly inequalities in access to care.

## Methods

The study is a mixed qualitative/quantitative study within an ethnographic framework described in detail elsewhere [13–15].

### Quantitative data

The quantitative data were based on a two-stage randomised procedure in which 40–50 patients with diabetes were selected from two PHCCs from each of the 24 regions of the country. PHCCs that hold medical consultations at least four days per week were included (n=567). Patient data were extracted from manual medical records. A maximum of 50 patients with diabetes were randomly selected per health centre. Data concerning patient characteristics, visits to the health centre, process of care, outcomes of care and prescribed treatment were extracted from the manual patient records. Process of care refers to whether a test had been recorded in the previous 12 months and was based on recommendations from the Tunisian national program [12], namely: Assessments of fasting glucose, blood pressure, weight, total cholesterol, creatinine, foot examination, cardiovascular examination, electrocardiogram (ECG), eye examination and HbA1c. Outcome of care refers to the result of the test, based on the national program recommendations [12]: Levels of fasting glucose, blood pressure, total cholesterol, body mass index and HbA1c. The outcome variables are acknowledged to be intermediate and not long term; it is not possible at present to identify long-term outcomes, such as complication and mortality rates, in the Tunisian setting.

Demographic variables, treatments prescribed and process and outcome data were compared using the chi-squared test for categorical variables and ANOVA for continuous variables. “Access to care” variables were compared between genders using logistic regression to adjust for confounding variables (age, type and duration of diabetes). Analyses were performed using Epi-Info and SPSS software packages.

### Qualitative data

Three purposively sampled PHCCs were selected for the qualitative aspects of the study. Purposive sampling involves developing a framework of variables based on the researcher’s practical knowledge of the research area, the available literature and evidence from the study itself [16]. The three PHCCs were selected based on variables that emerged from previous work and prior evidence from the study itself (health professional motivation, milieu and organisation of the health centre) [15]. Participant observation was undertaken weekly at the PHCCs for six months each by one of the authors (HA) who took a predominantly observing role, though acknowledging the role of the researcher as a research instrument as highlighted in reflective ethnography [17]. Observations and discussions pertinent to the research were recorded in fieldnotes. HA also spent designated time each week of the 5 year study reflecting on the data, and all constructive reflections were recorded in the fieldnotes. The potential influence of HA as a non-Tunisian, male, medical doctor was made explicit and incorporated into the study.

Fifteen focus groups (twelve consisting of patients and three of paramedical personnel), ten semi-structured interviews of clinicians and key informants and forty structured interviews of patients were undertaken at the three health centres. Subsequent to the finding from the quantitative work and participant observation that there was a large disparity in the numbers of men and women attending PHCCs for diabetes care, participants in the interviews and focus groups were asked specifically why they thought more women than men attend PHCCs for diabetes care.

Interviews and focus groups were audiotaped and later fully transcribed and simultaneously translated from Tunisian Arabic or French into English. Fieldnotes of relevant informal discussions were also recorded at the 48 randomly selected PHCCs used in the quantitative data selection.

The processes of sampling, data collection and data analysis were continuous and iterative. A content analysis [17] was conducted using the software computer program NVivo to systematically code and classify data into factors that influence care according to the predetermined categories of patient, health professional and organisational factors. The frequency that each factor was coded was noted in addition to the source (i.e. doctor, paramedical staff, patient, researcher or other). The reliability and validity of the data analysis and interpretation were ensured by the use of respondent validation, triangulation, prolonged engagement in the field, a clear audit trail and a reflexive approach [17, 18]. Respondent validation involved presenting and discussing a summary of the interviews of health professionals with the interviewee at a later date; additional comments were included in the transcripts. The quantitative data were collected in 2003/4 and includes data from 2000 to 2002; the qualitative data collection took place from 2003 to 2006.

## Results

### Quantitative data

The full study included 2160 patients with diabetes from 48 PHCCs with a mean age of 59.9 years (standard deviation = 14.1) and mean duration of diabetes of 8.4 years. Nearly all patients (94%) had type 2 diabetes. Approaching two-thirds (61.1%, n=1319) of patients attending PHCCs for diabetes care were women. Patient characteristics, including treatments prescribed, are shown in Table 1 .

Table 2 focuses on attendance and access to care differences between the genders. Women attended more frequently and were more likely to arrive at their appointment on time. However, the time until their next given appointment, as chosen by the doctor, was significantly longer. Women were also less likely to have their care recorded in the new disease-specific medical records.

**Figure 1:**
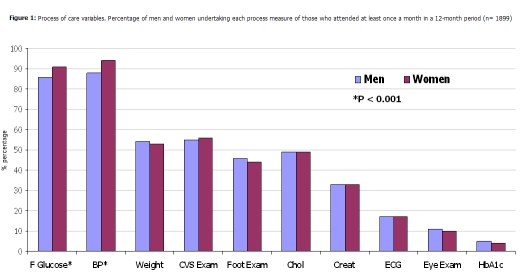
Process of care variables. Percentage of men and women undertaking each process measure of those who attended at least once in a 12-month period (n=1899)

Process and outcome of care data are shown in Figure 1 and Table 3 , respectively. Women were significantly more likely to have the two most commonly undertaken measures performed (fasting glucose and blood pressure); no other differences were found in the process measures. Women had significantly higher levels of systolic and diastolic blood pressure, total cholesterol and body mass index but lower mean creatinine levels than men.

### Qualitative data

A total of 15 paramedical personnel, 5 men and 10 women, participated in the staff focus groups and 40 patients, 25 women and 15 men, in the patient focus groups. The semi-structured interviews of clinicians comprised 6 women and 4 men and the structured patient interviews 27 women and 13 men. Respondents reported reasons for both men attending PHCCs less often and women attending more often ( Table 4 ). The most frequent reason given for men attending more was work commitments, related to the fact that PHCCs in Tunisia are only open in the morning for medical consultations: *“But the men, many of them work, most of them work, and they cannot come in the morning because they leave work at 6pm, the whole day they are working from 8am until 6pm…They have to stay at their work. Therefore, the health centre is not accessible. (Female doctor, semi-structured interview, Centre A). “I took a day off to come to the hospital today but they have just phoned me twice from work asking me to come in, so I have switched off my mobile phone”. (Male patient, focus group, Centre B)*

Another common perception was that men were less interested in looking after their own health than women: *“He said they (men) work and they don’t bother coming; they don’t bother about their illnesses until they die…” (Fieldnotes from discussion with a nurse, centre C)*

Other explanations offered were practical (men may attend other health care facilities such as occupational, private or secondary care), financial (men could afford to pay for private health care) and behavioural (men did not like attending primary care health centres and usually treated themselves). The health centre was often described as a ‘female domain’ and even equated with a female version of a man’s coffee shop. *“We discussed why there were more women than men at the health centres. They (2 male doctors) immediately blamed it on women over-attending and said it was a cultural problem; in the past women went to the well to draw water and that’s where they would gather to talk. Now there is nowhere to go so they come to the health centres. It’s cheap or free, they meet their friends, you can hear them chatting and laughing in the waiting room. They come too often. Sometimes they laugh and joke and then look ill when they go and see the doctor…. Dr. T said the men are ashamed to come and wait in the health centre since the centres are full of pregnant women or kids with their mothers; the centre is not a place for men – it is the territory of women.” (Fieldnotes from informal discussion with 2 primary care doctors)*

Men were observed to stand at or near the door, or sit at the back of the waiting room. They would often register and then leave, returning for their appointment. When asked, male patients admitted that they preferred to register and then wait outside until their turn rather than wait in the waiting room, which was full of women. In contrast, respondents felt that women tended to over-attend PHCCs. Patients and paramedical personnel, but not doctors, considered that women had increased morbidity, particularly due to stress and obesity. *“But for women, all their problems are in their heads… women have all the problems… they are more ill and more tired… even when their husbands die, they have to look after everything... Men don’t have many problems…” (Female patient, focus group, Centre A)*

There was a commonly held perception that women were “iller”, had more psychological problems and were more sensitive to their health needs: *“… most women are at home... they go to the hospital even to have their blood pressure checked… Women consult more than men, for all outpatient consultations, not just for diabetes and hypertension. And they have more psychological illnesses as well. There are the hypochondriacs; they sit at home all day and they feel everything and then their head hurts. So they go to the hospital. Even if they just feel a little light-headed they go to the hospital. Not like men..” (Male doctor, semi-structured interview, Centre B)*

In addition, respondents felt that women tended to over-attend, for social and insignificant reasons:

***
Nurse 1 (male):** Well look. Women by nature like to come and complain to doctors. They are more ill, they come to the hospital (health centre) more for many reasons, such as giving birth, and on top of that women by their nature like to attend. Men are more rational. Perhaps because of their work or because they are busier… this is for the majority of illnesses, not for diabetics only…
*
***
Interviewer:** And do you all think the same?
*
***
Nurse 2 (female):** Yes, for a woman her time is always spent at home. She always wants to come [to the health centre], especially those that don’t have the means for leisure activities. She attends and gathers and chats with her friends… this holds for the majority of women…(Focus group of staff, centre A)
*

The interview data revealed a clear-cut distinction in explanations of differential patterns of attendance for men and women, where women attended more for social and psychological reasons and men attended less for work-related reasons. Nevertheless, tensions or contradictions in, especially, women’s speech revealed a subordinate level of explanation. This is much like the ‘contradictory discursive framework’ noted in accounts of male health given by doctors and nurses in the Midlands area of the UK [19]. Researchers there found that doctors and nurses routinely deployed dominant discourses of masculinity to both critique and pander to the stereotype of male behaviour with consequences for patient care. In our case, the ‘contradiction’ was apparent especially in the speech of women when explaining their own attendance patterns. For example, following the usual explanation for attendance disparities which downplayed women’s actual health problems ( *“But for women, all problems are in women’s heads”* ) there was sometimes a moment of recognition that that there were real stresses and work loads that produce women’s illnesses, psychological or otherwise ( *“women have all the problems they are iller more and more tired and everything even when their husbands die, they have to look after everything”* ).

Some women patients were more emphatic, one declared about women that, *“Her responsibilities are many and she faces many hardships. They (men) could not stand them”* . Nonetheless, the dominant explanation remained that women’s problems were in their heads and their visits to health centres socially motivated. In addition, the significance of the PHCCs as places where women with few resources can pass some valuable leisure time was stressed by some women while underplayed by health professionals. As such, it would appear some respondents (especially women) recognized the structural, socio-economic and real health reasons for attendance rather than attributing the trend to psychological failings or trivialized social motivations, but that this recognition was subordinated in their discourse to the culturally dominant explanation.

## Discussion

We have demonstrated a number of disparities in the care of men and women with diabetes in Tunisian PHCCs. In particular, a dual but related problem emerges from the data: more women than men attended for diabetes care and yet women did not get the same level of risk factor control as men. The qualitative data revealed a widespread perception that women over attended while men under attended. A number of local explanations for this disparity have emerged, which inform our analysis of the impact of gendered beliefs on diabetes care in Tunisia.

### Quality of care

Differences in basic patient characteristics between men and women were to be expected in the Tunisian setting and have been shown in other surveys [20]. The difference in prescribed medications may be due to the higher prevalence of hypertension amongst women and the higher proportion of men attending with type 1 diabetes. The reason for the latter is unclear. It may be that men with type 2 diabetes are less likely to attend for care thus increasing the proportion of men attending with type 1 diabetes.

We found that women were less likely to have their care recorded in the new disease-specific medical records and their information was less complete; this has implications for care as use of these records has been linked to quality of care indicators [21]. Conversely, women were more likely to have their fasting glucose and blood pressure measured, the most commonly undertaken variables, which may be a consequence of women attending more frequently.

Women have significantly higher levels of systolic and diastolic blood pressure, total cholesterol and body mass index, confirming previous studies in Tunisia showing higher risk factors among women [20]. Lower serum creatinine levels are found in women due to lower rates of creatinine production from a smaller amount of muscle mass [22]. In general, these findings would support work from other parts of the world where gendered inequalities in diabetes care have emerged in some, but not all, areas of care [4–6].

### Access to care

Significant gender differences were found in areas related to access issues. Women attended more often and more regularly yet the time period until their next appointment, chosen by the doctor, was significantly longer. This may suggest some positive bias towards men with diabetes. Alternatively, this may be attributable to potentially higher complication rates in men thus requiring more regular consultations. The most striking disparity found in our study is the high female to male ratio of patients attending for diabetes care. Over 60% of patients attending health centres for diabetes care are women, despite the fact that the prevalence is only slightly higher in women than men [11]. Some, but not all, surveys in the primary care setting from other countries show a gender disparity in attendance rates but rarely as pronounced as our finding [6, 7, 10]. Women in Arab countries have been shown to attend physicians for all medical conditions more often than men although this difference diminishes after adjusting for economic and morbidity factors [8]. This concurs with a study conducted by the World Health Organization in 1998 that indicated that men are less inclined to engage in help-seeking behaviours [9]. It is important to acknowledge that the differences in attendance rates presented are to the public sector PHCCs, and men may disproportionally attend private and secondary care facilities, as indeed suggested by some of the participants themselves ( Table 4 ).

### Gendered beliefs and care

The present study offers a number of possible explanations for the gendered disparity in attendance and care revealed by the quantitative findings. Most participants considered that men were under represented at primary health care facilities. Reasons given for men not attending were practical, financial and behavioural. In contrast, there was a perception that women attended health care facilities more often than required. Participants perceived women to be “iller” and requiring more care. Reasons cited for the excess morbidity in women were stress, family and home responsibilities, and increased obesity. The underlying causes of disparities in care can be understood as the interplay of beliefs about the different “natures” of men and women, gendered domains and values and the perceived role of economic factors. As noted by other researchers, institutional settings such as health centres establish a discursive environment in which “normative parameters” exist for the performance of men’s and women’s identities [19, 23]. In the case of Tunisian PHCCs explanations and perceptions expressed by participants constituted a normative discursive environment that tended to attribute differences in care to men’s economic importance to the community and family, which ultimately was seen to derive from their “nature” as men, while women’s perceived over-attendance was attributed to their ‘nature’ as women who therefore attended for social and psychological reasons.

The normative discourses become entrenched because people who attend and work at the PHCC sites re-inscribe the centres and the types of activities undertaken there as gendered, which then structures beliefs about men’s and women’s “natures”. For example, reasons for women’s supposed increased morbidity were located in their minds, or in their “natures” as women. Such beliefs include the notion that women are “iller”, hypochondriacal, suffer from psychological or stress-induced problems, and need to socialize and chat. Furthermore, the types of “illness” cited – child birth, stress – were accorded less significance than men’s apparently real health issues for which they did not attend. As such, women were contradictorily classed as “more sick” and “not really sick”. Consequently, attending the PHCCs reinforces the belief that illness is synonymous with femaleness.

In contrast, beliefs about men’s “nature” might preclude them from seeking care. This would correspond to empirical research supporting the belief that men in general are reluctant to seek help from health professionals [24]. Although subject to cultural variation, findings from studies linking masculinity, culture and health stress that institutionalized cultural beliefs about male behaviour have consequences for health, especially where men are socialized to project strength and shun displays of emotion and other signs of vulnerability [25]. While not generalizable to all cultural contexts, this gender ideology is common to the circum-Mediterranean region [26, 27]. Additionally, although it has been noted that men as gendered subjects in the Arab world have rarely been the focus of sustained attention [28], Arabists and Islamist scholars have stressed similar ideals of masculinity in the Middle East and North Africa [28–31], although with important regional and local differences [29].

The existence of female and male domains has been noted widely, and especially in the region [30]. Some participants described health centres as a female domain where women congregated to socialise. Historically, PHCCs in Tunisia were introduced for maternity and child care only. Combined with the continued predominance of women, this strongly suggests that they are a female gendered domain for patients, explicitly contrasted to the male domain of work. Attending the PHCCs may in fact be perceived to be emasculating, much in the way suggested for infertility clinics in the Muslim world [32]. Work may thus be over-emphasized as a reason for non-attendance of men to counter-act this effect, meaning that poor attendance is as much about gender identity maintenance as it is about economic practicalities. As such, the assertion that “I was phoned twice from work” is about being recognized as, and feeling like, a male gendered subject within a purportedly female domain. Moreover, even though the perception that men seek care at private clinics needs to be formally evaluated, what is significant to our analysis is the perception that this is the case; whether true or not, indicating a connection between masculinity, work and wealth.

Similarly, other patterns in the data may be attributable to men’s identity maintenance. A suggestive example is presented by the discrepancy in attendance of men with type 1 versus type 2 diabetes. Type 1 diabetes may be sufficiently grave that men must seek treatment, thus posing less of a threat to men’s identities, whereas avoiding care for type 2 diabetes has less serious immediate health ramifications and therefore may be part of an affirmation of masculine identity.

The perception of men’s and women’s different “natures” was shared by all participants and reveals a negative valuation of women’s perceived over-attendance and a positive valuation of men’s under-attendance. There is a value hierarchy: perceived reasons for men not attending (work, money, lack of regard for health) appear to be valued more highly. The contradictory nature of the discourse on gendered patterns of attendance and health means that even when women’s illnesses are recognized as the result of stress and the responsibility of running a family, that knowledge is frequently undermined by or used to justify dominant gendered beliefs and practices.

In summary, by locating men’s reasons for not attending in economic practicalities and women’s perceived over-attendance in socio-psychological failings or weaknesses one set of reasons is given greater value and the other less. This may contribute to continued poor attendance for men and poorer care for women.

## Conclusion

A particular strength of this research is that it is part of a large, nationwide, multi-method study from a North African Arab country and thus the results are more likely to be transferable to similar countries than findings from the Western world, given differing historical and cultural experiences. It is important to note, however, that the qualitative work in particular is exploratory and the hypotheses proposed require formal evaluation.

The areas of apparent gender inequality need to be addressed in order to improve the quality of care of patients with diabetes in primary care. In particular, the possible explanations for the disparity in attendance require evaluation, and interventions to remove barriers to men attending PHCCs need to be developed. Both the distinction between perception and actual practice and recognizing the importance of a culturally specific system of gendered values are crucial in determining underlying reasons for gendered discrepancies in care. As such, it is important to recognize that questions of identity are significant factors in judging effective public health strategies [23]. In agreement with Seymour et al [19], the discursive frameworks revealed are as much instances of gendered practice as they are neutral reports of men’s and women’s behaviour.

Future measures would need to “defeminise” the centres as well as value women’s hardships and reasons for attending more. One possible course of action would be to have more flexible opening hours, such as opening the PHCCs for some period during the afternoon or evening. An alternative would be to offer separate gender clinics, separated by location or time. However, providing hours or locations that do not clash with men’s work lives, while important, may not be enough to reverse the trend, the roots of which run deeper and impact all levels of men’s health care, not only diabetes. Whichever strategy is adapted its implementation will require a careful consideration of local gendered discourses, beliefs and practices.

## Figures and Tables

**Table 1:** Clinical and demographic characteristics of the study subjects

** Characteristic **	** Data available **	** Men **	** Women **	p-value *
** Study subjects **	** 2160 **	** 841 (38.9%) **	** 1319 (61.1%) **	** x **
Age (years)	2109	58.0	60.9	<0.001
Duration of diabetes (years)	1469	8.5	8.7	0.58
Type 1 diabetes	2160	91 (10.8%)	43 (3.3%)	<0.001
Positive family history (%)	1311	261 (51.0%)	445 (55.7%)	0.11
Married (%)	1487	524 (86.3%)	604 (68.6%)	<0.001
Illiterate/less than primary school level education	1025	162 (40.6%)	487 (77.8%)	<0.001
Free care given due to low income	1589	64 (10.2%)	128 (13.3%)	0.060
Smoking history	1223	202 (38.5%)	25 (3.6%)	<0.001
Alcohol consumption	1106	57 (12.2%)	11 (1.7%)	<0.001
Past history of CVD	1273	27 (5.4%)	78 (10.1%)	0.004
Past history of RD	1229	32 (6.6%)	41 (5.5%)	0.50
Past history of LD	1195	38 (8.0%)	63 (8.8%)	0.71
** Treatment **				
Diet only	2160	27 (3.2%)	68 (5.2%)	0.041
Oral hypoglycaemic agents	2160	679 (80.7%)	1179 (89.4%)	<0.001
Insulin	2160	191 (22.7%)	219 (16.6%)	<0.001
Anti-hypertensive agents	2160	345 (41.0%)	742 (56.3%)	<0.001
Lipid-lowering medication	2160	117 (13.9%)	219 (16.6%)	0.105

* Chi-square test for categorical variables and ANOVA for continuous variables. CVD: Cardiovascular disease, RD: renal disease, LD: Lipid disorder

**Table 2:** Access to care variables

** Factor **	** Men (n=841) **	** Women (n=1319) **	* p-value
Number of visits in preceding 12 months **	3.65	3.75	0.07
Mean time until next appointment (days)	81.62	84.58	0.033
Consultations >2 weeks late (%)	27.7%	23.3%	0.082
Disease-specific medical records used (%)	89.3%	84.8%	0.08
Completion of new records (score of 12 variables *** )	7.11 ± 4.22	6.68 ± 4.27	0.014

* p-value using logistic regression with sex as the dependent variable and the factor in question plus age and health centre entered as explanatory variables.

** Excluding patients who did not attend at all in the 12-month period.

*** The 12 variables documented in the patient demographic section of the disease-specific medical records.

**Table 3:** Outcome of care variables

** Outcome **	** n **	** Mean **	** IQ range **	** Men **	** Women **	p-value *
Fasting glucose (mmol/l)	2071	10.2	3.7	10.1	10.3	0.07
SBP (mmHg)	2060	139.1	23.3	136.3	140.8	<0.001
DBP (mmHg)	2059	80.5	10.1	79.5	81.1	<0.001
Total cholesterol (mmol/l)	1520	4.9	1.4	4.7	5.1	<0.001
Creatinine (μmol/l)	1027	85.0	14.0	90.1	81.8	<0.001
BMI (kg/m ^ 2 ^ )	819	27.9	5.7	26.3	29.1	<0.001
HbA1c (%)	171	8.9	3.0	8.7	9.0	0.55

* Using ANOVA, IQ: Inter-quartile, SBP: Systolic blood pressure, DBP: Diastolic blood pressure, BMI: Body mass index

**Table 4:** Reasons cited by participants for gender disparity in attendance at PHCCs

** Reason given **	** Total **	** Doctors **	** Staff **	** Patients **
** Men under-attend **	** 45 **			
They work and cannot attend the PHCC	16	5	5	6
Not interested in looking after their health	12	1	4	7
Attend private institutions	9	4	0	5
Attend other institutions	3	0	1	2
Attend occupational health doctors	2	1	1	0
Fear of PHCCs	1	0	1	0
Treat their illness themselves	1	0	1	0
Because of shortage of medicines	1	0	0	1
** Women over-attend **	** 49 **			
Women are more stressed	12	0	2	10
Like coming to the PHCC for social reasons	9	2	5	2
Women are generally “iller”	8	0	3	5
Women are more obese	7	0	3	4
Women come for insignificant reasons	5	1	1	3
Women have nowhere else to socialise	5	2	2	1
Attend for psychological reasons	2	2	0	0
More sensitive to their health needs	1	1	0	0
